# UFL1, a UFMylation E3 ligase, plays a crucial role in multiple cellular stress responses

**DOI:** 10.3389/fendo.2023.1123124

**Published:** 2023-02-10

**Authors:** Qiang Jiang, Yongsheng Wang, Minghui Xiang, Jiamin Hua, Tianci Zhou, Fanghui Chen, Xiaoyang Lv, Jinming Huang, Yafei Cai

**Affiliations:** ^1^ Department of Animal Genetics, Breeding and Reproduction, College of Animal Science and Technology, Nanjing Agricultural University, Nanjing, China; ^2^ Institute of Animal Science and Veterinary Medicine, Shandong Academy of Agricultural Sciences, Jinan, China; ^3^ Department of Respiratory Medicine, Nanjing Drum Tower Hospital Affiliated to Medical School of Nanjing University, Nanjing, China; ^4^ Department of Hematology and Medical Oncology, Emory University School of Medicine, Atlanta, GA, United States; ^5^ International Joint Research Laboratory in Universities of Jiangsu Province of China for Domestic Animal Germplasm Resources and Genetic Improvement, Yangzhou, China

**Keywords:** UFL1, Ufmylation modification, ER stress, genotoxic stress, oncogenic stress, inflammation

## Abstract

The UFM1 conjugation system(UFMylation)is a novel type of ubiquitin-like system that plays an indispensable role in maintaining cell homeostasis under various cellular stress. Similar to ubiquitination, UFMylation consists of a three-step enzymatic reaction with E1-like enzymes ubiquitin-like modifier activating enzyme5 (UBA5), E2-like enzymes ubiquitin-fold modifier-conjugating enzyme 1(UFC1), and E3-like ligase UFM1-specific ligase 1 (UFL1). As the only identified E3 ligase, UFL1 is responsible for specific binding and modification of the substrates to mediate numerous hormone signaling pathways and endocrine regulation under different physiological or pathological stress, such as ER stress, genotoxic stress, oncogenic stress, and inflammation. Further elucidation of the UFL1 working mechanism in multiple cellular stress responses is essential for revealing the disease pathogenesis and providing novel potential therapeutic targets. In this short review, we summarize the recent advances in novel UFL1 functions and shed light on the potential challenges ahead, thus hopefully providing a better understanding of UFMylation-mediated cellular stress.

## 1 Introduction

Post-translational modifications (PTMs), a modifications system of protein amino acids introduced to the substrate in an enzymatic or nonenzymatic manner, plays a crucial role in increasing proteome diversity and affects virtually all cell biological pathways in eukaryotic cells (1). As one of the most well-known post-translational modifications, modification of ubiquitin (Ub) proteins or ubiquitin-like (UBL) proteins is a rapid and effective regulation to maintain cell homeostasis during cellular stress responses (2, 3). Dysfunction of the modification process triggers a diverse range of cell process dysfunction, including cell cycle progression, signal transduction, protein translation, DNA damage response, antiviral response, and autophagy (1–4).

### 1.1 Core components of the UFM1 conjugation system

**Figure 1:**
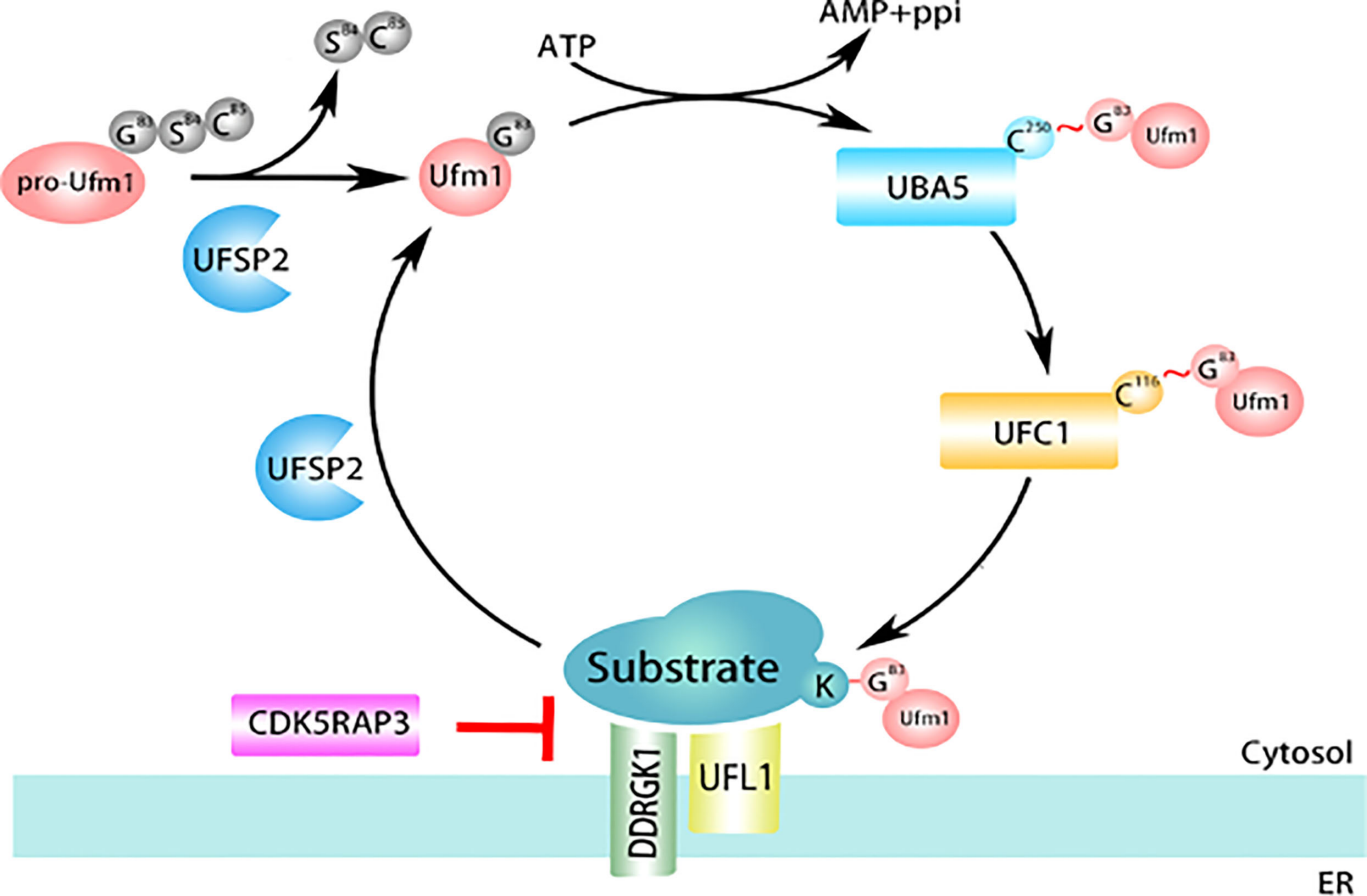
The Ubiquitin-Fold Modifier 1 (UFM1) Conjugation Pathway.

The ubiquitin-fold modifier 1 (UFM1) conjugation is a newly-identified ubiquitin-like system (5). Similar to ubiquitination, the UFMylation process consists of a three-step enzymatic reaction. At the beginning of the modification process, UFM1 precursor (proUFM1, 85 amino acids) is processed to cleave the last two residues (Ser84 and Cys85) by UFM1-specific proteases (UFSP2) to generate mature UFM1 with Gly83 exposed in C-terminal, which is essential for the UFM1 cascade reaction and conjugation to the substrate. Whereafter, mature UFM1 is activated by forming a high energy thioester bond between the exposed Gly83 and Cys250 of UBA5 (E1 enzyme), then transferred onto the catalytic Cys116 of UFC1 (E2 enzyme). Subsequently, UFM1 was transported from UFC1 to its substrates mediated by UFL, the only E3 ligase identified in the UFMylation system. At the end of the process, UFM1 can be removed from substrates by UFSP2 for recycling (5–9) (**Figure 1**).

### 1.2 UFMylation system E3 ligase-UFL1

Thus far, UFL1(alternate name as RCAD, KIAA0776, NLBP, and Maxer) is the only identified E3 ligase reported with the UFM1 conjugated function (7). The structural characteristics of UFL1 protein are composed of 794 amino acids with an approximate molecular mass of 90kDa. Different from other E3 ligases, UFL1 does not characterize any specific functional domain, such as the HECT and RING domains, but its N-terminal region can link UFC1 and DDRGK1 (9). At the subcellular level, UFL1 and DDRGK1 are mainly located on the endoplasmic reticulum membrane in the cytoplasm, where they are anchored to the ER membrane by the N-terminal signal peptide of DDRGK1 (6). Additionally, the N-terminal region of UFL1 is highly conservative in different species, indicating that UFL1 plays a role in the evolution of species.

### 1.3 The specific substrates of UFMylation

According to previous research, under the ufymlation system, UFL1 performs its biological functions mainly by modifying UFM1 to the substrates. The biological processes involved include hematopoiesis, liver development, kidney development, DNA damage repair, autophagy, and intestinal homeostasis (**Table 1**).

**Table 1:** The role of UFMylation in pathology and physiology.

Substrates	UFMylation sites	Involved biological processes	Possible mechanism	Reference
DDRGK1	Lys267	Hematopoiesis	DDRGK1 deficiency cause ER stress and activation of UPR, which led to cell death of hematopoietic stem cells.	(10)
		Autophagy	DDRGK1 control lysosomal function to regulate autophagy.	(11)
		Antibody synthesis	DDRGK1 modulate UPR to promotes plasma cell development and ER expansion.	(12)
		Trypsin synthesis	DDRGK1 prevent ER stress-induced apoptosis in protein secretory cells.	(13)
ASC1	Lys324, 325, 334, 367	Breast cancer	ASC1 UFMylation promote ERα transactivation in breast cancer development.	(14)
RPL26	Lys132, 134	Protein biogenesis	RPL26 UFMylation control the co-translational protein translocation into the ER.	(15)
		Hematopoiesis	RPL26 UFMylation cope with increased secretory flow during erythroid differentiation, and ultimately influence hemoglobin production.	(16)
		ER phagy	RPL26 UFMylation repress the unfolded protein response *via* IRE1a in ER-phagy.	(17)
Histone H4	Lys31	DNA damage repair	Histone H4 UFMylation activates ATM signaling and maintenance of genomic integrity.	(18)
MRE11	Lys282	DNA damage repair	MRE11 UFMylation is required for the MRN complex formation and ATM activation, mediate DNA repair and genome integrity.	(19)
CDK5RAP3	Not available	Liver development	CDK5RAP3 loss caused ER stress and activated IRE1α and PERK signaling pathways.	(20)
		Intestinal homeostasis	CDK5RAP3 deficiency led down regulation of key transcription factors Gfi1 and Sox9 in Paneth cell fate specification.	(21)
P53	Lys351, 357, 370, 373	Tumour suppress	P53 UFMylation maintain p53 protein stability and tumour-suppressive function.	(22)

#### 1.3.1 DDRGK1

DDRGK1, also known as UFBP1, c20orfll6, and Dashurin, was the first identified UFMylation substrate (6). However, further study show DDRGK1 is much more like an adaptor protein for UFL1 anchored at the ER (9). The DDRGK1 protein consists of 314 amino acid residues and contains an N-terminal transmembrane segment, an extended helical region named NTR (N-terminal region), and a winged helix (WH) domain. The UFMylation site of DDRGK1 occurs at the Lys267, where it mediates protein-protein interactions and forms multiprotein complexes. Previous studies show that DDRGK1 forms a scaffold-type ligase complex with UFL1, thus binding to UFC1 to promote aminolysis and transfer UFM1 onto substrates. However, DDRGK1 also plays a role in a UFMylation-independent way in autophagy, such as acting as an ER surface adaptor for UFL1 rather than a UFMylation substrate to promote ER-phagy (17). Additionally, our research shows that DDRGK1 promotes autophagy by impairing the mTOR pathway and decreasing autophagy degradation by inhibiting autophagosome-lysosome fusion, indicating the dual effect of DDRGK1 is indispensable for autophagic degradation (11).

#### 1.3.2 ASC1

Activating signal cointegrator 1 (ASC1), also named thyroid hormone receptor interactor 4 (TRIP4), was previously identified as a transcriptional co-activator of estrogen receptor α (ERα). By forming a dimeric complex with 17β-estradiol, ERα translocates to the nucleus and recruits transcriptional coactivators, binding to ER-responsive elements (EREs) and activating gene expression downstream. Thus, ERα is a prominent growth factor in breast cancer. Yoo et al. reported that ASC1 can be ufmylated at the sites of Lys69, Lys324, Lys 325, Lys334, and Lys367, and ufmylated ASC1 enhances the ability of ERα recruited transcription co-factors, increasing the target genes expression, thereby affecting the intracellular homeostasis and ultimately tumor formation (14).

#### 1.3.3 RPL26

More recently, the Ribosomal Protein L26 (RPL26), a component of the large ribosomal subunit responsible for the synthesis of proteins in the cell, was identified as a primary target of UFM1 conjugation (15). In the progress of protein translocation, ribosome stalling active the attachment of UFM1 to RPL26, and catalyzed by UFL1/DDRGK1/UFM1 enzyme complexes, RPL26 is UFMylated at the site of Lys132 and Lys134 on ER membrane. Strikingly, further biochemical analysis confirmed that by promoting the translocation-arrested ER protein to lysosomes, RPL26 UFMylation regulated the degradation of stalled nascent chains *via* a proteasome-independent way (16). In metazoan, UFMylated RPL26 forms a protein quality control mechanism, safeguarding co-translational protein translocation into the ER, which is essential for protein biogenesis.

#### 1.3.4 Histone H4

Histone H4 is a core component of the nucleosome, which tightly wraps DNA and achieves the nucleosome’s compact chromatin structure. However, histone H4 has tails that project from the nucleosome. By post-translational modifications to the tails, histone H4 influences all DNA-based processes, such as transcription, chromatin compaction, DNA replication, and reparation. For instance, Histone H4 acetylation is essential for opening chromatin during replication, and histone H4 methylation regulates DNA replication by compacting chromatin. Recently, Qin et al. reported that the site of Lys31 on histone H4 was ufmylated by UFL1 following DNA damage. Ufmylated histone H4 is vital for amplifying ataxia-telangiectasia mutated (ATM) kinase activation, which orchestrates the DNA reparation and maintenance of genomic integrity (18).

#### 1.3.5 MRE11

Meiotic recombination 11 (MRE11) encodes a nuclear protein, which is one of the vital response proteins in DNA damage, functions in DNA double-strand break repair (DSBR) and other DNA damage response (DDR). MRE11 protein is the core of the MRE11-RAD50-NBS1 (MRN) complex, which acts as the early sensor and responder for locating DSBs. In the presence of the MRN complex, ATM is activated and recruited to the DSB site and triggers the downstream DDR pathway, tethering and resecting damaged chromosomes (23). Similar to Histone H4, MRE11 is UFMylated on the site of Lys282 induced by DSB. MRE11 UFMylation promotes MRN complex formation and recruitment to the DNA damage stripes, and defective UFMylation impairs ATM activation and genome stability (19).

#### 1.3.6 CDK5RAP3

Cdk5 activator p35-binding protein C53 (CDK5RAP3, also known as C53, HSF-27, IC53, LZAP, MST016) was initially identified as the binding protein of Cdk5 activator p35, CBP, and ARF. Previous studies show that Cdk5rap3, as a substrate adaptor for UFMylation, is essential for the growth, proliferation, and functional maturation of hepatocytes, loss of Cdk5rap3 in mice suffering liver hypoplasia, and prenatal lethality. The hepatocyte-specific Cdk5rap3 knockout mice displayed impaired lipid metabolism and hypoglycemia, leading to death after weaning (20). Additionally, intestinal epithelial cell (IEC)-specific Cdk5rap3 knockout mice displayed a nearly complete loss of Paneth cells and increased susceptibility to colitis, indicating the essential role of Cdk5rap3 in Paneth cell development and maintenance (21). Our research results show that CDK5RAP3, a novel nucleoplasmic shuttle or molecular chaperone, affects nucleoplasmic translocation and trimer formation of heat shock factor 1 (HSF1), involved in heat stress response regulation and mammary epithelial cells heat injury protection (24). These findings have demonstrated the role of Cdk5rap3 in both development and homeostasis.

#### 1.3.7 P53

TP53 is a tumor suppressor gene, which encodes a protein with a molecular weight of 53kDa, so the coding protein is named p53. As the most intensively studied tumor suppressor transcription factor (21), P53 function tumor suppressor and induces cancer cell growth arrest or apoptosis depending on many intrinsic and extrinsic factors of cells (25, 26). TP53 mutations were found in about half of human cancers, most leading to the different expression of mutant p53 protein, which acquired transforming activity (27, 28). The critical negative regulator of p53 is E3 ubiquitin ligase MDM2, which mediates the degradation of p53 ubiquitin-proteasome, keeping p53 protein at a low level (29, 30). When cells undergo a cancerous reaction, the p53 degradation pathway is restricted, accumulating p53 protein and initiating a series of downstream gene transcription. Liu et al. report that p53 performed ufmylated at the four sites of Lys351, Lys357, Lys 370, and Lys373. Because these Lys residues are also subject to ubiquitination, the UFMylation plays a crucial role in p53 stability maintenance (22).

## 2 The regulatory mechanism of UFL1 in cellular stress response

**Figure 2:**
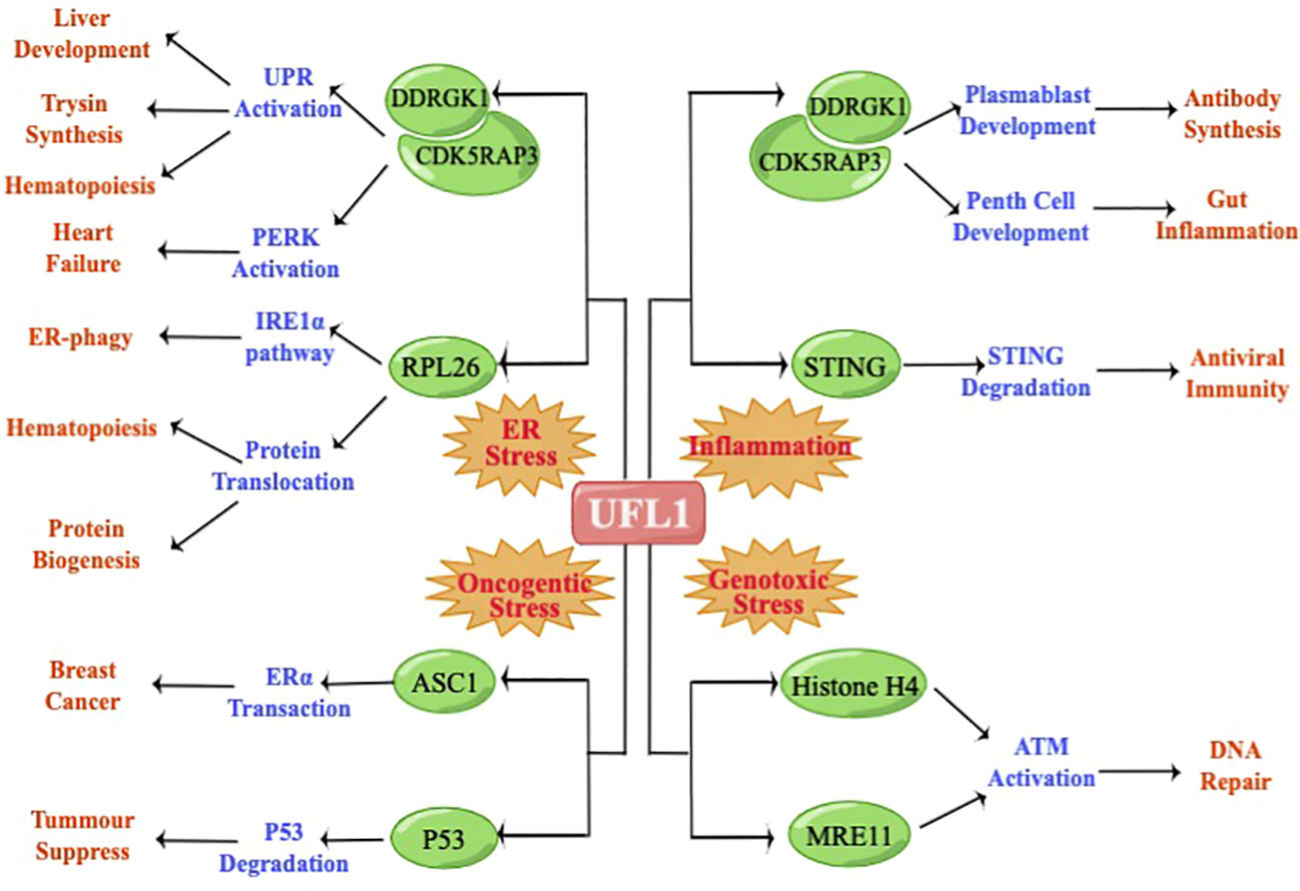
UFL1 regulate different physiological processes depending on the stimulus and the cellular background.

In order to further clarify the function of UFL1 in multiple stress, we summarized the substrates and regulatory pathways of UFL1 in cellular stress response, including ER stress, Genotoxic stress, Oncogenic stress and Inflammation (**Figure 2**).

### 2.1 ER stress

The endoplasmic reticulum (ER) is the central organelle responsible for the biosynthesis of cholesterol, steroids, and calcium homeostasis, regulating second messenger signaling (31, 32). However, endoplasmic reticulum homeostasis is dynamic homeostasis influenced by a variety of factors, such as inflammatory reaction, starvation, calcium depletion, and hypoxia, which can break the balance and impair the function of the endoplasmic reticulum, causing a large number of unfolded or misfolded proteins to accumulate in the process of protein synthesis and processing, eventually lead to activate ER stress (33, 34). Upon ER stress, cells actively evolve three protein quality-control pathways: unfolded protein response (UPR), ER-associated degradation (ERAD), and autophagy pathways (35). As the critical quality-control method, UPR regulates ER stress by four pathways: 1) UPR buffers fluctuations in unfolded protein loading by transmitting information about unfolded protein loads, inhibiting protein synthesis in the ER capsule to restore ER homeostasis (36). 2) UPR enhancing ER chaperone expression levels to upregulate the protein folding ability, including Dnajb9, ERdj4, Bip (also named Grp78), and PDIa3 (37–39). 3) UPR triggers the degradation of misfolded protein by the ERAD pathway (35). 4) When cells undergo irreversible ER stress, UPR induces cell apoptosis to eliminate damaged cells by enhancing autophagy (40). That indicates that ER stress-related mechanisms incorporate information about stimulus duration and intensity.

Based on the findings that the UFMylation system is highly enriched in the ER, it is a close relationship between UFMylation and ER stress. On the clinical side, ER stress induced by the dysfunction of UFMylation causes a series of diseases, including hematopoietic disease (10, 41, 42), heart disease (43, 44), exocrine pancreatic disease (13), type 2 diabetes (45), intestinal exocrine disease (46), schizophrenia (47), breast cancer (14). In the hematopoietic system, our research show that deletion of UFL1 and DDRGK1 causes defective erythroid development and embryonic lethality(E10.5–E13.5), severe lesions observed in fetal liver (FL), the erythroid differentiation fromcolony forming unit-erythroid (CFU-Es) to proerythroblasts blocked, ER stress elevated and UPR activated at the cellular level. Knock out either UFL1 or DDRGK1 in bone marrow cells and hematopoietic stem cells, increase the expression of ER chaperones ERdj4 and Bip, elevated phosphorylation of eIF2α, XBP1 mRNA alternative splicing, and the upregulated of cell death markers (Bax, Noxa, Puma and DR5) (10, 41). These phenomena revealed that the pathogenesis of hematopoiesis abnormality might be ER stress and UPR induced by the deficiency of UFMylation, especially considering hematopoietic stem cells are very susceptive to ER stress (48). Bip is a primary molecular chaperone to fold proteins in the ER lumen, which is identified as an ER stress-specific marker that activated in the UPR pathway (49). The upregulation of Bip benefits nascent polypeptides folding correctly, degrading misfolded proteins, and maintaining calcium homeostasis in the ER (50).

UFMylation also functions in other ER stress susceptive secretory cells, for instance, pancreatic cells. The deficiency of UFL1 in pancreatic cells caused decreased Bip expression, which disrupted ER homeostasis. It led to trypsin activation and pancreatic acini death, indicating that the UFL1/BiP pathway is essential for the synthesis and secretion of pancreatic enzymes correctly (39). Another typical secretory cell is plasma cells which are responsible for secreting antibodies. UFL1 and other UFMylation system components are displayed high expression in plasma cells and maintain the development of plasma cells by suppressing the activation of protein kinase RNA-like ER kinase (PERK), which is one of the ER stress sensors. Deficiency UFMylation blocks the differentiation of B cells into plasmablast and impairs ER expansion in plasma cells, thus regulating humoral immune responses.

In heart disease, UFL1 cardiac-specific knockout mouse display increased expression of fetal cardiac genes, elevated fibrosis, and impaired cardiac contractility, ultimately developing into cardiomyopathy and heart failure. Mechanistically, UFL1 protects against cell death induced by ER stress by regulating ATF6 signaling and calcium cycling in cardiomyocytes, which is proven to be crucial for cardiomyocyte contractility and potentially impaired by depletion of UFMylation, indicating the essential role of UFL1 for cardiac homeostasis by regulating the function of ER (44).

In summary, the dysfunction of the UFL1 impairs the quality control system in the endoplasmic reticulum, induces ER stress and UPR, breaks cellular homeostasis, and disrupts the autophagic pathway, ultimately inducing cell apoptosis. By forming a UFL1/DDRGK1/UFM1/C53 tripartite receptor complex with the ER lumen, UFMylation regulates the degradation of proteins and senses proteotoxic stress, eventually evolving into an ancient quality control pathway that bridges lysosomes autophagy with ribosome translocation-arrested protein quality control in the ER (17, 42).

### 2.2 Genotoxic stress

Stable genomic DNA is necessary for the accurate transmission of genetic information. However, genotoxic stress includes DNA single-strand breaks, double-strand breaks, base damage, and the numerous alterations that block DNA replication, impairing genome stability and cell viability. To ensure genome integrity and prevent tumorigenesis, cells have evolved to derive a set of DNA damage response (DDR) collaborative networks, such as cell cycle checkpoint system, DNA repair system, chromatin recombination, gene expression regulation, and other signal pathways (51–55). The protein ATM is an upstream DDR kinase, functions as an apical activator of the DNA damage response, and controls signaling and the DNA repair network. Under normal homeostatic conditions, ATM exists in the dimeric or multimeric form without activity. Meanwhile, ATM is activated under genotoxic stress by MRN complex (MRE11-RAD50-NBS1), recruited to the DNA damage sites, and activates repair function (56, 57).

Up to now, only two UFMylation substrates (MRE11 and histone H4) have been identified under genotoxic stress, and both function in ATM activation (18, 23). Wang et al. found that UFL1/DDRGK1 physically interacts with the MRN complex, and the interactions are dynamically modulated by DNA damage. Further studies revealed that the Lys282 of MRE11 was modified by UFM1, which DSBs induce. MRE11 UFMylation promotes MRN complex formation and the recruitment of MRE11/NBS1 to the DNA damage sites. Defective UFMylation compromises impair MRN complex formation and ATM activation induced by DNA damage, which ultimately maintains the stability of the cell genome under genotoxic stress. In line with these findings, the same phenomenon was observed in another substrate, histone H4. Up on DNA damage, UFL1 is recruited to the DSB site by MRN complex and ufmylated histone H4 at Lys31. Subsequently, UFMylation of H4 modulates ATM activation by enhancing the recruitment of Suv39h1 and Tip60. Interestingly, although the Lys31 site was also reported to be ubiquitinated by Cul4A (E3 enzyme complex), which then enhances histone H4 transcription (58), the ATM activation is independent of different histone H4 Lys31 ubiquitination or gene expression. In addition, ATM phosphorylated UFL1 at the S462 site, enhancing the E3 ligase activity of UFL1, forming a positive and dynamic feedback loop to boost ATM activation.

In general, UFL1 functions as an ATM signaling regulator, playing a vital role in DDR signaling under genotoxic stress

### 2.3 Oncogenic stress

The molecular epidemiological datasets show that the UFMylation pathway may have dual effects under oncogenic stress. Specifically, genetic variation of the UFMylation components (UBA5, UFC1, UFL1, DDRGK1, and UFM1) has been identified in various tumors in The Cancer Genome Atlas (TCGA) database, such as breast carcinoma, hepatocellular liver carcinoma, ovarian serous cystadenocarcinoma, lung adenocarcinoma, esophageal carcinoma, lung squamous cell carcinoma, uterine corpus endometrioid carcinoma, bladder urothelial carcinoma, and sarcoma (59). In addition, UFL1 and DDRGK1 have been reported to suppress the NF-kB signaling pathway, and DDRGK1 knockdown inhibited cell proliferation and invasion in U2OS cells (60). However, the expression of UFL1 or DDRGK1 was closely associated with the overall survival rate of patients with renal clear cell carcinoma (22).

Based on the present findings, two substrates were identified explicitly involved in cancer development. ASC1 is the first substrate identified in breast cancer development (14). Mechanistically, in the presence of estrogen hormone, ERα binds to ASC1 instead of UFSP2, leading to ASC1 UFMylation, which is mediated by UFL1 and DDRGK1. Upon UFMylation, ASC1 may act as a scaffold protein, potentially enhancing the coactivator recruitment, including p300 and SRC1, which is essential for ERα binding to EREs, thus upregulating the ERα target gene expression, such as pS2, cyclin D1, and c-Myc. Since breast cancer is highly ERα sensitive and positive, ASC1 UFMylation promotes breast cancer cell growth and tumor formation. Another substrate is p53, the most critical tumor suppressor, which can be ufmylated by UFL1 (22). As a central hub pathway that governs various critical cellular processes, p53 is a short-lived protein under normal homeostatic conditions (61). However, p53 is stabilized primarily by a post-translational modification to act as a tumor suppressor under oncogenic stress. The primary degradation mode of p53 is ubiquitination regulated by mouse double minute 2 homologue (MDM2), the principal E3 ligase for p53 degradation. However, UFL1 can bind to the C-terminal of p53, and the region is where MDM2 binding. Further research confirmed that UFL1 competes with MDM2 binding to p53, and depletion of UFL1 promoted the interaction of p53 with MDM2. Subsequently, UFMylation of p53 competes with its ubiquitination, counteracting its proteasome degradation, thus maintaining the stability of the p53 protein. In line with the vitro result, the mouse xenograft assay experiment shows that depletion of either UFL1 or DDRGK1 results in tumor size and weight increase, suggesting that UFL1 and DDRGK1 function as tumor suppressors by modulating p53 stability.

Collectively, the research results of substrate demonstrate the multiple effects of UFMylation in tumorigenesis depending on different cellular contexts.

### 2.4 Inflammation

Inflammation is a central feature of an effective immune response, which prevents the invasion of pathogenic microorganisms and other foreign material. The activation of immune response depends on pattern recognition receptors (PPRs), including Toll-like receptors (TLR), nucleotide-binding oligomerization domain-like receptors (NLR), and RIG-I-like receptor (RLR), to discern pathogen-associated molecular and activate downstream signal-pathways (62, 63). Among these, the stimulator of interferon genes (STING), a critical adaptor of IFN regulatory factor 3 (IRF3) and nuclear factor kappa-B (NF-κB) signal pathways, is crucial for effective innate immune responses (64). Tao et al. reported that UFL1 regulates the cGAS-STING signal pathway by targeting STING. Deletion of UFL1 causes the decreased expression of interferon-β (IFN-β) and interleukin-6 (IL-6). Mechanistically, UFL1 inhibits TRIM29 (ubiquitination E3 ligase) from interacting with STING, thereby inhibiting K48-linked ubiquitination and proteasomal degradation of STING, indicating that UFL1 is an important factor in maintaining STING stability and antiviral function. Interestingly, in this research, UFL1 functions in a UFMylation-independent way (65). In humoral immune responses, plasma cells are in charge of secreting antibodies, which play a critical role in the immunoglobulins and antibody responses. Zhu et al. reported that DDRGK1 UFMylation is dispensable for developing plasma blasts. CD19-specific DDRGK1 knockout blocks the differentiation of B cells into plasma blast, causing a significant reduction of antibodies activated with lipopolysaccharide (LPS) (66).

In addition, both UFL1 and UFM1 were found to be up-regulated in inflammation induced by LPS (12, 46). Elevated expression of UFL1 relieved the LPS-induced ER stress and apoptosis by regulating the TLR4/NF-κB pathway, thus alleviating the inflammatory response and cell damage in bovine mammary epithelial cells. Depletion of UFM1 induced increased ubiquitination and degradation of IκBα, thereby inhibiting NF-κB p65 nuclear translocation in resident peritoneal macrophages. Several recent research results show that the UFMylation system is indispensable in gut inflammation. Specifically, ablation of any of them (UFL1/DDRGK1/CDK5RAP3) in intestinal epithelial cell (IEC)-specific knockout mice displayed disappear of Paneth cells, leading to an imbalance of intestinal microbiota and increased susceptibility to colitis. At the cellular and molecular levels, the loss of DDRGK1 or CDK5RAP3 causes increased ER stress and the activation of UPR, disintegrating rough endoplasmic reticulum (RER) and abnormal zymogen granules in mature Paneth cells (21, 46).

Taken together, these results provided unambiguous evidence for the essential role of UFMylation maintenance of congenital immunity and gut inflammation.

## 3 Conclusions and perspective

Overall, this review discussed the roles of UFL1 in multiple cellular stress responses and provided some examples that illustrate how the UFMylation acts as a dynamic post-translational modification system for maintaining homeostasis. It is proved that the UFMylation system components (UFM1/UFL1/DDRGK1/CDK5RAP3) are part of different regulatory modules that coordinate the fine-tuning of important homeostatic processes in multiple cellular stress.

However, with limited knowledge, elucidating the underlying molecular mechanism of UFMylation remains challenging. Here we list some examples of questions that need to proceed for future research.

1) how can UFL1 be manipulated to enable appropriate stress responses and restore homeostasis? To explain this, we surmised that UFL1 functions by recruiting adaptor and signaling proteins, especially the transcription factors, to assemble the signaling platforms perceived stress sensors, therefore modulating the amplitude and kinetics of downstream responses.

2) Is any other stress responses that UFL1 involved in? The stress response is the protective mechanism of cells against changes in the external environment. UFL1 has been proven activated in including but not limited to ER stress, genotoxic stress, oncogenic stress, and inflammation. However, how UFL1 performed in other environmental conditions, such as heat stress, oxidative stress, variable pH, calcium deficiency, and viral infection, is still mysterious. Therefore, exploring UFL1 function in different physiological or pathological stress responses is an important direction for future research.

3) For UFMylation progress itself, still needs to be better understood. Although recent studies have identified the components involved in the UFMylation system, the regulatory relationship between them is puzzling. For instance, CDK5RAP3, a substrate adaptor that can form an integrated complex with UFL1, restricts UFL1/DDRGK1 E3 ligase activity. However, some phenotypes are similar between CDK5RAP3^-/-^ and UFL1^-/-^ mice, which raises the question of whether there is an unknown regulatory mechanism among the UFMylation components.

Taken together, UFMylation is a newfound and attractive modification system. Appreciation of the UFMylation molecular mechanism of maintaining homeostasis under multiple cellular stress, as well as its feedback regulation, sheds light on stress-induced disease pathogenesis and provides novel therapeutic targets for treating relevant diseases.

## Author contributions

QJ: writing original draft and editing. YW: writing-original draft. MX: investigation. JinH: investigation. TZ: Data sorting. XL: Data sorting. FC: review and editing. JinH: editing and supervision and funding acquisition. YC: editing and supervision and funding acquisition. All authors contributed to the article and approved the submitted version.
